# Historical institutionalism and policy coordination: origins of the European semester

**DOI:** 10.1007/s40844-023-00253-z

**Published:** 2023-04-15

**Authors:** Wen Pan, Madeleine O. Hosli, Michaël Lantmeeters

**Affiliations:** 1 Sichuan University, Chengdu, China; 2 Leiden University, The Hague, The Netherlands; 3 Vrije Universiteit Brussel (VUB), Brussels, Belgium

**Keywords:** European semester, Multilateral surveillance, Historical institutionalism, Legal-historical analysis, Path dependence, EU economic and fiscal governance, Institutional stickiness, B20, N0, N940, P520

## Abstract

Using historical institutionalism as a theoretical foundation, this paper explores whether multilateral surveillance and policy coordination under the European Semester (ES), introduced in the aftermath of the global financial crisis of 2007–2008, was based on a path-changing or a path-dependent mechanism. Following a legal-historical analysis going back to the Maastricht Treaty, we demonstrate that the ES in its institutional form was not the result of a crisis-induced critical juncture, but rather of a gradual, path-dependent historical evolution which kept the balance of power relations in the European Union (EU) broadly unchanged. With this, we offer a critique of literature on the post-crisis European economic governance framework, which sees an increase in the influence of supranational actors, notably the European Commission. Focusing on the ES, our paper puts forward an alternative view, grounded in the theory of historical institutionalism, arguing that the balance of power relations has broadly stayed the same. The conclusion is based on a legal-historical analysis of 12 indicators, which help to understand the evolution of the ES.

## Introduction^1^

In the wake of the pressures to the Eurozone emanating from the 2007–2008 global financial crisis, important steps in European economic governance were initiated, leading to an actual new European Union (EU) architecture of post-crisis governance. To understand the nature of these measures, it is important to explore how respective EU approaches in economic and fiscal governance developed, from the earlier preventive arm of the Stability and Growth Pact (SGP) to the European Semester (ES), which induced a yearly scheme of monitoring, planning and if needed, correction. Did the ES represent an incremental change (i.e., path dependency) rather than a substantial reform (a critical juncture of a new path) to EU economic and fiscal policy coordination? Authors have held different views on this aspect. We explore this topic based on process-tracing and in-depth document analysis.

The European sovereign debt crisis, triggered by the global financial crisis, displayed the challenges related to lacking fiscal cohesion and stability among the Eurozone states. The ES was established as a reaction in January 2011 as part of a larger package of reforms. The scheme was created and implemented in accordance with the ‘preventive arm’ of the SGP and its remit later expanded with the implementation of the ‘six pack’ and ‘two pack’ reforms. In essence, the ES represented a new, reinforced, annual response mechanism to facilitate the coordination of national budgetary and economic plans of EU member states. It was adopted by the EU Council of Economic and Financial Affairs (ECOFIN) on 7 September 2010 and codified into EU law in 2011 (Council of the European Union 1997, 2011a).

A shared budgetary surveillance timeline for Eurozone member states was introduced on 30 May 2013 based on Regulation 473/2013 (Council of the European Union 2013). Moreover, in 2014, the European Commission conducted a review of the application and effectiveness of the SGP, which, in 2015, led to the informal adoption of more flexibility in its application (European Commission 2014a, 2015; Seikel 2016; Zeitlin and Verhercke 2018). Next to more flexibility, gradually, more attention was devoted in the ES context to social rather than purely economic-fiscal policy considerations (Zeitlin and Verhercke 2018).

Some scholars have argued, or implied, that the implementation of the ES has strongly affected the balance of power among EU member states and European institutions, with an increase in influence of the supranational actors (e.g., Dehousse 2016; Jones et al. 2016; Nicoli 2020). In this sense, the introduction of the ES represented a ‘critical juncture’ (or deviation from an earlier path taken), partially due to a ‘failing forward’ mechanism of European integration induced by the crisis. Others were more cautious, however, and discovered less of a departure from earlier provisions (e.g., Schimmelfennig et al. 2015; Börzel 2016; Genschel and Jachtenfuchs 2018). While our paper agrees with many aspects of earlier work, it re-evaluates the critical junction claim, applying concepts derived from historical institutionalism (HI) (e.g., Thelen 1999; Mahoney 2000; Mahoney et al. 2016) and using process-tracing to discern some relevant historical-legal developments leading up to the ES. With this, by situating the ES into its historical context, we aim to judge whether its introduction represented either a substantial or incremental change to then ongoing patterns of EU fiscal and economic governance. The choice for the application of HI is appealing not least because it adds to rival claims between intergovernmentalists and neo-functionalists in European integration theorizing, by constituting a ‘golden mean’ between explanations based on such competing grand theories of European integration. HI more generally has the potential to overcome the impasse of the dichotomous debate between these main approaches (e.g. Pollack 1996, 2009; Rosamond 2000, 2010, 2013).

HI reflects one of the three schools of new institutionalism. The other two—rational choice and sociological institutionalism—may be seen as the polarized ends of a spectrum, whereas HI is located in the middle. In this position, HI benefits from insights offered by both other schools of thought and therefore, incorporates both rational choice and sociological elements in its theorizing (Aspinwall and Schneider 2001: 2–5; Rosamond 2010: 110).

Incorporating HI propositions, we will present two main hypotheses guiding our analysis. We then test their validity by offering a legal-historical account of respective developments based on process-tracing. With this approach, we present an alternative perspective and supplement to other comparative methods. In a nutshell, process-tracing aims to uncover casual mechanisms suggested by theoretical approaches (George and Bennett 2005: 153). If the outcome of a case studied is in line with the theoretical expectations, a causal mechanism may be in effect. Process-tracing can uncover and identify the causal relationship between an independent and a dependent variable, focusing on ‘the unfolding of an event over time’ and examining ‘the chain of events’ (Panke 2012: 129), answering both ‘why’ and ‘how-come’ questions (Panke 2012: 136). This is what we endeavor to do, focusing on the ES as an important element in the development of European economic post-crisis governance, preceding the latest, significant steps taken in the framework of the NextGenerationEU project.

The selection of the ES as a case is based on a ‘most likely’ research design: only a single case will be studied in-depth. Causal mechanisms of EU integration are expected to ‘most likely’ be found in new steps of economic and fiscal policy coordination, such as the ES. While conducting our analysis, we are aware that the ES was only one element in a broader approach to EU post-crisis economic governance.

Based on an analysis of legal-historical processes, we will demonstrate that the ES in its institutional form was not something radically new, rather a result of a gradual, path-dependent historical evolution. With this, our study adds to the current literature on the European economic post-crisis governance regime by an explicit focus on a historical perspective and related changes in EU legal-institutional terms.

The paper is structured as follows. Section two provides an overview of HI’s propositions on path dependence and critical junctures. It discusses the three-step analytical model (T0–T1–T2) and based on this, formulates two main hypotheses. Section three presents the actual case study and the results obtained by our analysis. Thereafter, section four adds a critical look at our findings based on a wider interpretation of the ES in view of instruments such as the Excessive Deficit Procedure (EDP) and the Excessive Imbalances Procedure (EIP), capturing changes and adaptations to the original ES measure. Lastly, section five summarizes the main findings of our paper and concludes.

## Theoretical framework and hypotheses

HI can be seen as a variation of New Institutionalism, which itself is a revised version of Traditional Institutionalism or ‘Old Institutionalism’. There are multiple variations of New Institutionalism. According to Peters (1999), however, they have four common features related to  core aspects of an institution: (1) it is a structure, formal (e.g. legal framework) or informal (e.g. network or shared norms); accordingly, between actors, “some sort of individual patterned interactions […] are predictable”; (2) it is characterized by some stability over time; (3) it affects individual behavior based on its formal or informal features and constraints; and (4) it has “shared values and meaning” among its members. Based on these four shared features, Peters distinguishes six variations of New Institutionalism.

By comparison, Hall and Taylor (1996) offer a categorization based on three institutionalisms, namely the large clusters of rational choice, historical and sociological institutionalism. This categorization is especially helpful to distinguish the major features of ‘institutionalist’ approaches.

Applied to EU studies, rational choice institutionalism (RCI) assumes that actors involved in EU decision-making behave rationally and employ strategies to realize their preferred outcomes; consequently, the EU is generally analyzed based on principle-agent models (Aspinwall and Schneider 2001: 7). As such, RCI research on the EU mainly focuses on rationalist approaches to legislative procedures, the implementation of rules, measures and policies, and the analysis of the European Court of Justice (ECJ). In comparison, Sociological Institutionalism (SI) applied to the EU focuses on identifying normative and cultural mechanisms constructing or constraining national behavior. It studies how identity itself influences state interests and behavior, as well as the international normative structures underpinning them. SI touches upon a wide variety of topics such as domestic-EU relations, regionalism and European integration, policymaking of member states, European citizenship and EU enlargement. Given the differences in their fundamental assumptions and research foci, RCI and SI are often viewed as two polar opposites of a spectrum.

HI tries to overcome this polarization by placing itself in the middle (Rosamond 2010: 110); it highlights how prior institutionalist arrangements and commitments condition further action, set limits on possible options, and lead actors to redefine their interests (Pierson 1996, 1998; Aspinwall and Schneider 2001: 10). It assumes that “institutions reflect the complex and unique structures that influence both the interests of actors and action arenas” (Sitek 2010: 570). In other words, “history creates context, which shapes choice” (Aspinwall and Schneider 2001: 10).

According to Hall and Taylor (1996), HI has four distinctive features. First, it uses a broad conception of the relationship between institutions and individual actions. Second, it emphasizes power relations and asymmetrical power distributions during the establishment and operation of institutions; a given institution provides some interest groups with “disproportionate access to the decision-making process” (Hall and Taylor 1996: 941). Third, it views institutional development through the lens of ‘path dependence’ and ‘unintended consequences’. Finally, it sees institutions as one factor in a causal chain, resulting in political outcomes in the end.

HI accounts for both rational choice assumptions of self-interested utility seeking actorness and sociological assumptions, such as socioeconomic development and the propagation of ideas and beliefs (Hall and Taylor 1996: 942). As such, HI studies of EU integration can be close to RCI when institutions are seen as power neutral, but simultaneously, they can be close to SI when emphasizing cultural factors and institutional power implications to social groups (Aspinwall and Schneider 2001: 11–12). With this, HI also incorporates elements of intergovernmentalism and constructivism in the analysis of European integration.

HI emphasized difficulties in terms of controlling institutional evolution and the need to take an ‘evolving’ rather than a ‘snapshot’ view of European integration (Pierson 1998: 30). Important to this approach is that the EU can be valuably explored in a historical context, as its political development unfolds over time. Secondly, its process and current developments are “embedded in institutions—whether these be formal rules, policy structures, or social norms” (Pierson 1998: 29; 2000: 264–65).

According to the path dependence perspective within HI, sequences of decisions matter and past choices exert an influence over today’s decisions by making certain alternatives appear more attractive (for an analysis applied to the United Nations Security Council, see Hosli and Dörfler 2019). Once a course of action is chosen it will be difficult to diverge significantly from it, as the *status quo* holds precedent and a drastic change requires a large investment of political resources (Lelieveldt and Princen 2011: 42). Core mechanisms of path dependency are ‘sunk costs’, ‘increasing returns processes’ and ‘self-reinforcing or positive feedback processes’. According to Pierson (2000: 252) “the relative benefits of the current activity compared with other possible options increase over time” while “the cost of exit—of switching to some previously plausible alternative—rise”. Hence, “the probability of further steps along the same path increases with each move down that path” and “preceding steps in a particular direction induce further movement in the same direction” (Pierson 2000, 252). These previous steps “make reversal very difficult” (Pierson 2004: 10). Moreover, “increasing returns” are self-reinforcing as “long movement down a particular path will increase the costs of switching to some previously forgone alternative” (Pierson 2000: 261). They constitute “positive feedbacks” as “institutions and policies generate incentives for actors to stick with and not abandon existing institutions, adapting them only incrementally to changing political environments” (Pollack 2009: 127).

Path-dependent decision-making can be problematic, however, as ‘form’ determined by path dependency is often preferred over ‘functionality’. Hence, path dependency can result in positive, but also negative externalities, such as ‘unintended consequences’. Negative repercussions imply that institutions need to change, but change would require a relatively large investment of political resources. Investment of such resources will often be higher than the costs of negative externalities, causing a continuation of the *status quo*.

However, path dependence can be interrupted by ‘critical junctures’. These are unforeseen drastic changes in the internal or external environment, such as crises. Only when these occur, can path dependency undergo drastic change, as it legitimizes the allocation of the needed large investment of political resources. In such instances, one witnesses ‘branching points’, where radically different patterns of behavior can be initiated and different decisions are possible, detached from the courses determined by ‘path dependent’ processes (Hall and Taylor 1996: 942; Pierson 2000: 263). It is important to keep the two terms, ‘path dependence’ and ‘critical junctures’ distinct, however, as it is the former that lays the foundations for the latter, but this is not interchangeable. Critical junctures then can have ‘lasting consequences’ (Pierson 2000: 263).

The path dependency framework seems particularly applicable to see whether the ES constituted a fundamental change (critical juncture) compared to earlier attempts at European economic and fiscal policy coordination. This matters not least when comparing such earlier steps to the more recent one of the establishment of the NextGenerationEU scheme as a reaction to the potential economic crisis effects of COVID-19.

A fundamental change, in general, is more in accordance with intergovernmentalist theorizing on European integration, as agreement between governments is core to new steps taken. By comparison, incremental change is closer to neo-functionalist perspectives on integration, interpreting steps potentially as ‘spillover effects’. Both can be applied to economic aspects of European integration.

A critical juncture, in the European integration context, could also be accompanied by shifts in terms of the allocation of competencies and power, both in terms of ‘vertical’ relations (national-supranational) and those among EU member states (national-national). To explore whether the ES might have created such power re-allocation effects, we formulate a first hypothesis: the ES is the result of a critical, path-changing juncture that altered national-supranational and/or national-national power relations (H1).

If evidence is found for H1 in our case, e.g. by constraints on national governmental competencies in the sense of potentially ‘unintended consequences’, HI’s arguments on crises as causes of critical junctures gain support. By comparison, if we fail to find evidence for H1 in our historical-legal exploration, then the ES is likely to rather represent a path-dependent development based on earlier provisions (notably the SGP).

We will resort to legalist criteria on national-supranational and national-national competences and power distributions as the gauge to assess such potential ‘critical junctures’ or ‘new paths’. In this context, a helpful conceptual elaboration of the path dependence framework is the ‘three-step analytical model’, embodying core ideas and mechanisms of HI.

Pierson (1996, 149; 1998, 49), while exploring the ‘path to European integration’, has made crucial contributions to the ‘three-step analytical model’ relevant to HI. In essence, T1 is the period of ‘grand bargains’ by member states, resulting in considerable gaps in national government control, and leading to an altered context for T2. There are four variables that notably characterize the changes from T0 to T1: (1) shifts in domestic circumstances, (2) the occurrence of micro-level adaptations (‘sunk costs’), (3) accumulated policy constraints and (4) heavily discounted or unintended effects. Clearly, (liberal) intergovernmentalist thinking is close to such explanations, notably as domestic interests and power constellations affect intergovernmental negotiations (the ‘grand bargains’). The shift from T1 to T2 then indicates a change in the internal—i.e., variables contributing to T1—or external context, forged by altered member state preferences, changed bargaining strength of EU states, or altered powers of other actors. These factors together explain the institutional and policy outcomes after the ‘grand bargains’ taking place at T2. Pierson argued that decisions arising from ‘the short-term preoccupations of institutional designers’ could “undermine (…) the long-term control of member state governments” and lead to widespread unanticipated consequences (1998: 56). Such explanations deviate from liberal intergovernmentalist thought notably by their focus on unintended consequences of governmental action.

Institutional arrangements and increasing ‘sunk costs’ in European integration (i.e., ever-increasing costs of exiting from existing, supranational, institutional arrangements) are then making any reversal both difficult and unattractive in practice. The logic here is not only driven by a concern with institutional constraints at a ‘macro level’, but also by activities of societal actors at the ‘micro level’, incrementally building up their vested interests in EU policies.

Drawing on contributions of various authors using HI and incorporating elements of RC, Hix (1999, 2005) conceptualized the three-step analytical model of European integration (T0–T1–T2) as follows:

“At time T0, a set of institutional rules is chosen or a policy decision is made (by the member state governments), on the basis of the structure of existing preferences. At time T1, a new structure of preferences emerges under the conditions of the new strategic environment: the changed preferences of the member states, the new powers and preferences of the supranational institutions, and the new decision-making rules and policy competences at the European level. And, at time T2, a new policy decision is adopted or a set of institutional rules is chosen.” (Hix 1999: 16)

According to Hix’s conceptualization of the process, at the first stage (T0), national governments are in control. Decisions taken then ‘lock’ the integration process into a particular ‘path’. Hence, “the decision taken by the member states at T2 is very different from that which they would have taken if they had faced the same decision at T0” (Hix 2005: 17). The shift from T0 to T1, by comparison, results from changed national preferences due to an altered internal or external environment. According to Hix, three variables in the shift from T0 to T1 contribute to institutional adaptation: changed preferences of relevant actors, new powers and preferences of supranational institutions and new decision-making rules or policy competences at the EU level. The policy outcomes then occur at T2. Both in the shift from T0 to T1 and from T1 to T2, however, can unintended consequences occur as a result of actors’ imperfect information to predict changing preferences at T1 or policy outcomes at T2. Such unintended consequences can take the form of disproportional shifts in governmental policy competencies towards the supranational level (Hix 2005: 17), altering the balance of influence between EU states and supranational institutions. This could also be relevant for the case of the ES.

Similar causal chains have been proposed by other researchers, such as Drezner (2010). His approach to path dependency is as follows:

“At time t, a set of rules R is codified. These rules help to shape and reinforce the preferences of the salient actors. At time *t*+1, the cost of switching away from R is somewhat higher. With each iteration, the reinforcement between actor preferences and the rules that bind them make it increasingly unlikely that R will be changed endogenously” (Drezner 2010: 794).

Drezner’s description highlights the potential unlimited ‘iteration’ and ‘reinforcement’ of rules R via the time stages *t*, *t* + 1, *t* + 2, *t* + 3, …, *t* + *n*. However, the analysis of rules R can also be divided into sequences of T0–T1–T2. In his interpretation, Drezner proposes three variables that contribute to a shift from T0 to T1 (or in his model, from t to *t* + 1): (1) higher switching costs from the rules codified at t (comparable to Pierson’s ‘sunk cost’ explanation), (2) the reinforcement of links between actors’ preferences and the rules that bind them, and (3) an decreasing likelihood that the rules codified at t are changed endogenously. Pierson’s and Hix’s T0–T1–T2 model stress the evolving relationship between states and supranational institutions on institutional rules and policymaking outcomes over time, while Drezner’s focus is more on the rules themselves and the underlying factors driving the reinforcement and ‘stickiness’ of established rules and policies.

Hix’s T0–T1–T2 model and Drezner’s *t*, *t* + 1, *t* + 2, *t* + 3, …, *t* + *n* approach can hence be viewed as two separate but complementary approaches capturing path dependency mechanisms: Hix’s model emphasizes the historical and institutionalist context to explain how a path-dependent mechanism works and evolves, whereas Drezner emphasizes the dynamics of path dependence themselves — i.e. the increasing costs of switching away from a path. Moreover, Drezner’s argument that “it (is) increasingly unlikely that R will be changed endogenously” (Drezner 2010: 794) implies that to break up the current chain of reinforcement and embark on a new path may require exogenous pushes (such as a crisis).

We will use Pierson’s T0–T1–T2 ‘path map’ as the baseline for our analysis but synthesize it with Hix’s approach to ‘new structures of preferences’ and Drezner’s ‘switching costs’. Accordingly, in T0 the decision is taken to create an institution. The institutional and policy outcomes are determined by interstate bargaining processes. The (intergovernmental) negotiation outcome will reflect actors’ preferences and bargaining power. A shift from T0 to T1, in this ‘combined version’, is dependent on a total of nine factors: (1) changes in domestic conditions, (2) micro-level adaptations (‘sunk costs’) or higher ‘switching costs’ from the rules codified at T0, (3) accumulated policy constraints, (4) heavily discounted future outcomes or ‘unintended effects’, (5) changed preferences of actors involved, (6) new powers and preferences of supranational institutions, (7) new decision-making rules and policy competences at EU level, (8) reinforcement between actors’ preferences and the rules that bind them, and (9) decreasing likelihood that the rules codified at T0 are changed endogenously.

The shift from T1 to T2 is then determined by (1) T1 dependent changes to preferences of relevant actors, (2) changing bargaining powers of these actors, and (3) influence and powers of other actors. This then leads to institutional and policy outcomes in phase T2.

The application of the three-step analytical approach, following the T0–T1–T2 path dependency model, hence focuses on institutional and policy evolution, with a core element being the EU intergovernmental ‘grand bargains’ needing unanimous approval and leading to Treaty revisions. Each round of the three-step model then reflects negotiation results of an Intergovernmental Conference (IGC). This incorporates conditions contained in Pierson’s model, while accounting for changed structures of preferences and new contexts in which policymaking is embedded (as argued by both Pierson and Hix). Each EU Treaty revision will be based on previously adopted Treaties and each Treaty change and expansion implies a potential of reiteration and reinforcement of initial rules, as suggested by Drezner. We will now aim to empirically explore such steps based on a historical-legal analysis.

The formulation and adoption of the SGP occurred within the framework of negotiations on the 1992 Maastricht Treaty (the Treaty on European Union, TEU) and as a result of the creation of European Economic and Monetary Union (EMU), which constituted a core economic and monetary development in European integration. Combining the approaches by Hix (1999, 2005), Drezner (2010) and Pierson (1996, 1998) discussed above, we now introduce a second hypothesis (H2), proposing that a path dependency mechanism occurred but based on specific conditions. Building on H1, H2 claims that the ES reflected a path-dependent development starting from the Maastricht Treaty, continuing over the Amsterdam (signed 1997) and Nice (signed 2001) Treaties and resulting in the provisions contained in the Lisbon Treaty (signed 2007). It has to be acknowledged, however, that there was an additional potential institutional step: the actual European Constitution (European Communities 2005). It was signed in Rome in 2004 but never entered into force, as it was rejected in referenda in France and the Netherlands and then led to the (more modest) Lisbon Treaty. We will hence not explicitly stipulate its provisions, which were similar, however, to those related to economic governance in the Treaty of Lisbon. It is the economic background of potential path dependency and effects of path dependency on economic outcomes in European integration that matter to our analysis, including H2.

H2 as a hypothesis can be seen as a heuristic, specifying causal factors to empirically analyze a potential path-dependent evolution of the ES. We will explore the validity of each of these 12 factors.

**Fig. 1:**
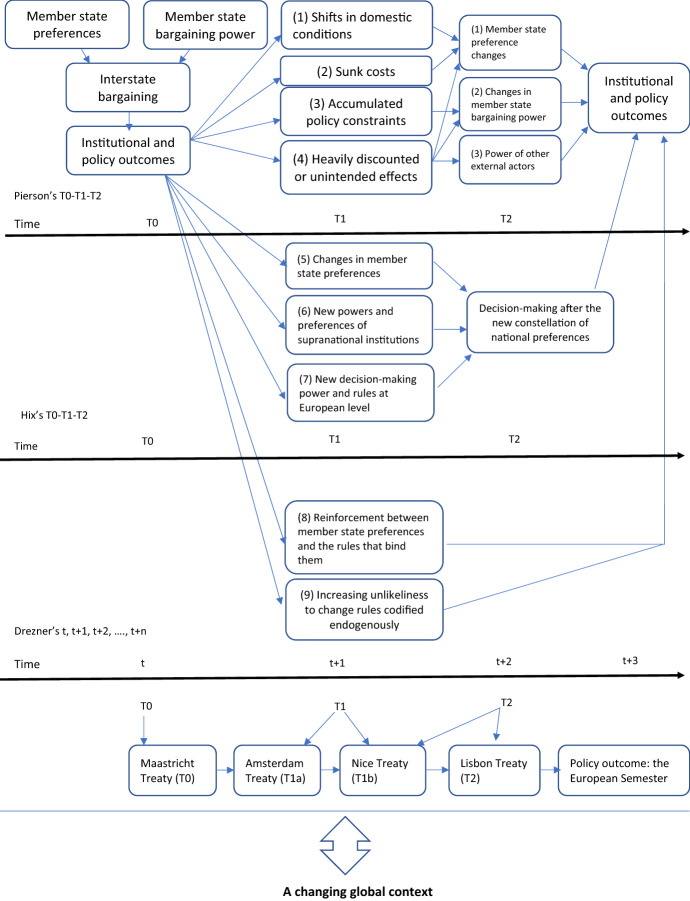
Adapted three-step analytical model: the European Semester matched to respective European Treaty reforms. T0 Treaty of Maastricht (TEU) 1992; T1a Amsterdam Treaty 1997; T1b Nice Treaty 2001; T2 Treaty of Lisbon, 2007. Sources: Adapted from Pan (2015, 2018), based on conceptualizations by Pierson (1996, 1998), Hix (1999, 2005) and Drezner (2010)

The overarching model underlying our analysis is presented in Fig. 1. The analytical factors—or indicators—summarize the different approaches as presented above. We integrate the respective stages and variables (boxes) and potential causal chains (arrows) to drive the analysis.

Accordingly, in Fig. 1, the Lisbon Treaty is categorized as T2, whereas the Maastricht Treaty (the TEU) marks T0. As a component of the intergovernmental agreements reached at T0, the SGP was to ensure budgetary discipline after the adoption of EMU and the single currency by Eurozone member states. The TEU not only stipulated three stages to realize EMU, but also specified the convergence criteria applying for member states to join EMU, with this “constitut(ing) the legal basis for the EMU and its new single currency” (Hosli 2005: 37).

Governments of EU member states conducted IGC negotiations, resulting in decisions on EMU and its new common currency, the Euro and respective steps to be taken, as spelled out in the Maastricht Treaty. Member state governments at that time—albeit having different priorities for EMU—can be assumed to have had control over the respective policies and outcomes. This included the creation of the SGP, to maintain stability within EMU and the creation of Stability and Convergence Programs (SCPs) aiming to coordinate and monitor national-level fiscal and economic behavior after the introduction of EMU. Between T0 (Maastricht Treaty) and T2 (Lisbon Treaty) is T1—two chronological intergovernmental ‘grand bargains’: T1a (Amsterdam Treaty) and T1b (Nice Treaty), both of which can satisfy interpretations of T1 in the framework of the HI three-phase model.

Synthesizing all factors shown in Fig. 1 provides the basis of the empirical exploration, offering concrete operationalizations for hypotheses one and two above. Accordingly, factors (1)–(9) for T1 and (1)-(3) for T2, resulting in a total of 12 indicators, constitute the analytical core elements of our study.

## The adoption of the European semester

Largely as a response to the European sovereign debt crisis, the ES was introduced in 2011 as a new coordination and surveillance mechanism monitoring national-level performance within the EU. It complemented initiatives such as the creation of the European Financial Stability Fund (EFSF) and later, the European Stability Mechanism (ESM). In essence, the ES is “[a] 6-month period each year when EU Member States’ budgetary, macroeconomic and structural policies are coordinated so as to allow these countries to translate EU considerations into their national budgetary processes and into other aspects of their economic policymaking” (European Stability Mechanism 2017).

The ES was not an instrument availing the EU of legally binding powers. In essence, it included three approaches to strengthen economic and fiscal policy coordination: monitoring of (1) national fiscal policies within the EU and (2) of their economies, and (3) preventing excessive macroeconomic imbalances. Overall, the annual ES procedure was divided into the ‘European semester’ in the first half of the year and the ‘national semester’ in the second half. This established a shared timetable for policy guidance and monitoring at the EU level with policy implementation at the national level. Though the semester cycle officially commences at the beginning of each year, the preparatory work starts at the end of the previous year. In November and December, the European Commission was to publish an Annual Growth Survey (AGS) and an Alert Mechanism Report (AMR), with the AGS stating EU policy priorities that member states should take into account when drawing up their own economic policies for the coming year and the AMR reviewing individual member state macroeconomic developments. Based on the AMR, the European Commission had the option to conduct In-Depth Reviews (IDRs) to identify potential high macroeconomic imbalances and make relevant policy recommendations to the member states concerned. In January and February, the Council of Ministers was to discuss the AGS, formulate orientations and adopt conclusions. The EP would then deliberate over the AGS and publish its own initiative report and issue its opinions on Employment Guidelines (EGs). In March, based on the AGS and the Council’s analysis and conclusions, the European Council was to provide policy orientations, which member states were to take into account when preparing their SCPs (budgetary policies) and National Reform Programs (NRPs) (policies promoting growth, employment and competitiveness). The European Commission could issue IDRs on macroeconomic imbalances and draft Country-Specific Recommendations (CSRs) to remedy such imbalances. In April, EU states were to submit their Stability and Convergence Programs (SCP) and National Reform Programs (NRP), while in May, the European Commission was to evaluate the submitted plans and issue draft CSRs. The Council in June would discuss the drafts and reach agreement (with possible amendments) on the final CSRs, requiring endorsement of the European Council in June. In the next month, the Council would adopt the CSRs, member states would begin implementing them (by taking the CSRs into account in their national decision-making processes regarding national budget and economic policies in the following year). Consequently, “governments, when submitting the draft budget to the national parliament, are expected to include policy recommendations by the Council and/or the Commission accompanied by an explanation of how these have been incorporated” (The Task Force Report 2010: 10).

Towards the end of each calendar year, a new cycle of the ES was to start again. The ES grew alongside other EU steps, most notably the two reforms made to the SGP as a consequence of the European sovereign debt crises: the ‘six-pack’ and the ‘two-pack’, providing a more complete regulatory framework. Moreover, in March 2011, ‘Euro Plus Pact’ commitments were integrated into the SCPs and NRPs checked in the framework of the ES. In December 2011, the ‘six-pack’, reforming the SGP *inter alia,* codified the ES into EU regulation (Article 2-a of Regulation (EU) No 1175/2011). It introduced a new dimension to EU policy coordination and surveillance aiming to prevent macroeconomic imbalances by the Macroeconomic Imbalances Procedure, MIP (Regulation (EU) No 1176/2011; Council of the European Union 2011b). It also reinforced the EP’s role by integrating an Economic Dialogue into the ES, providing it with the possibility to intervene in the ES at almost any point in time (Regulation (EU) No 1175/2011). In May 2013, Regulation (EU) No 473/2013—one of the ‘two-pack’ regulations—added an autumn counterpart to the spring semester procedure to check Euro member states’ budgetary plans and the Excessive Deficit Procedure (EDP) countries’ Economic Partnership Programs.

In essence, when created in 2011, the ES integrated different strands of economic policy coordination into a new single surveillance cycle, “bring(ing) together existing processes under the SGP and the Broad Economic Policy Guidelines (BEPGs), including simultaneous submission of SCPs and NRPs” (COM(2010) 526 Final 2010: 2). According to Regulation (EU) No 1175/2011, codifying the newly-invented policy coordination procedure, the ES includes BEPGs, EGs, SCPs, NRPs and surveillance mechanisms to prevent and correct macroeconomic imbalances. While these measures may seem technical, they all constitute important steps in the further development of the ES. To capture these developments, relying on official documents and additional literature when applicable, Table 1 provides an overview of differences between the situation before and after the introduction of the ES.

**Table 1:** The European semester and previous provisions compared Sources. Adapetd from Pan (2015, 2018), based on Council Regulation (EC) No 1466/97; Council of the European Union (2005); European Commission (2010a, b); Hallerberg et al. (2011); Delors et al. (2011); European Commission (2012); Hallerberg et al. (2012); European Commission (2014b)

Previous Provisions (SGP and Lisbon Strategy)	European Semester	Transformative Change in Practices?
SGP: agreed in 1997; introduced requirement that SCPs be submitted by all EU member states. Prescribed that the SCPs “shall be submitted before 1 March 1999. Thereafter, updated programs shall be submitted annually”. BEPGs and the EGs incorporated	Guidance and surveillance at EU level are synchronized; fundamental components of the ES, however, can be traced back to SGP; adoption of national budgetary and economic plans at the national level of EU states; change is largely from *ex-post* to *ex-ante* policy coordination. Old sanctions regime for euro area countries implemented	*No*
2005 Lisbon Strategy introduced a set of new and more powerful measures to steer and monitor economic policy reform: (a) Integrated Guidelines (IGs); (b) NRPs; (c) CSRs; (d) new open methods of coordination (OMCs). IGs, for the first time, combined the BEPGs and the EGs into a single document. Based on the IGs, each EU member state was to draw up a NRP, and due to the newly-invented CSRs, ‘[f]or the first time, policy advice covering the entire field of economic and employment policy was submitted to the European Council and the Council on a country-specific basis’	ES adopted the former coordination tools either as they were or in a somewhat reformed way; CSRs do not constitute legally binding obligations. If an EU member state fails to implement the CSRs within the given timeframe, it will not be fined or taken to Court; only policy warnings can be issued. Member states’ compliance with the CSRs still relies on (a) peer pressure, (b) market pressures, and (c) possible sanctions that fall under other separate mechanisms controlled by the European Council, such as the Excess Deficit Procedure (EDP) or the Excessive Imbalance Procedure (EIP). Accordingly, no actual change in competencies or transformative change in practices	*No*
In 2005, Council Regulation (EC) No 1055/2005 amending Regulation (EC) No 1466/97 adopted, introducing new elements into the SGP’s preventive arm; e.g. assessment of the long-term sustainability of public finances in the SCPs	In ES, competence distributions remain essentially unchanged; no legally binding decisions possible. EU member state governments are responsible for final implementation of CSRs, but cannot be forced to do so. Accordingly, no transformative change in practices	*No*
Before 2011, due to the existence of separate and different policy coordination procedures, ‘[t]here was no comprehensive view of the efforts made at national level, and no opportunity for Member States to discuss a collective strategy for the EU economy’	ES provides platform for collective strategy of EU member states.From 2011 onwards, the NRPs and SCPs have to be submitted simultaneously, following a common timetable and procedure, under a single mechanism. All synchronized policy coordination is now *ex ante*, as EU priorities and objectives enshrined in the AGS are issued ‘before the drawing up of the NRPs and SCPs and before the adoption of national budgets’. The new regulations contained in the ‘six-pack’ and the ‘two-pack’ introduce special provisions for the Eurozone countries aiming to further strengthen Eurozone economic governance. This leads to unbalanced governance patterns between the Eurozone and the non-Eurozone blocks within the EU. Decisions under the ES procedure are not legally binding for EU member states. Thus, both national-supranational and national-national power relations concerning EU budgetary and economic policy coordination remain constant and are similar to the preventive arm of the SGP (as agreed in 1997). Accordingly, there is no transformative change in practices	*No*

Exploring the various provisions related to the ES, based on the indicators outlined above, it can be stated that no fundamental changes were introduced with the introduction of the ES. It rather appears that the ES has resulted from detailed steps that can be captured by a path dependence process, originating in the SGP (notably its ‘preventive arm’). But as Table 1 also shows, according to this exploration, neither national-supranational nor national-national competences and powers changed by the introduction of new mechanism, providing further support for the assumption that there was no (crisis-induced) ‘critical juncture’.

This finding is in line with Amy Verdun’s (2015) more general historical institutionalist analysis of the EU crisis response, which she found was largely based on action by already existing institutions. Actors’ endogenous preferences, moreover, can be a result of path dependencies—a core concept of HI (Schimmelfennig 2018). National governments can be seen as having been the main actors in terms of policy response, but supranational level intervention remains critical for the actual policy enforcement (e.g., Falkner 2016). Moreover, as national government powers did not seem to get (further) constrained or reduced, the ES is not likely to have constituted an unintended consequence of the initial decisions taken through the SGP. However, the case study results as shown in Table 1 do reveal that the already existing cleavage of Eurozone compared to non-Eurozone EU states has been reinforced by both the SGP reforms and the introduction of the ES, with this accentuating the trend towards a two-speed EU. Responses to the European sovereign debt crisis, including more intensive fiscal and budgetary policy coordination may have reinforced a given North–South division within the EU.

HI’s assumption of the role of economic crises in triggering critical junctures, as our analysis demonstrates, does not seem to have been applicable in the case of the ES. Rather, our overview (Table 1) demonstrates the ‘stickiness’ of EU policy in terms of coordinating and monitoring mechanisms in terms of member state fiscal and economic behavior.

Hypothesis 2 started from the premise that that a path-dependent development began with the Treaty of Maastricht, continuing over the Amsterdam and Nice Treaties to the provisions incorporated in the Lisbon Treaty. Based on theoretical reflections, we assumed a range of causal factors to potentially have determined the process. To explore the validity of H2, Table 2 provides an overview of the detailed changes in regulations as incorporated into the various Treaty revisions.

**Table 2:** The Evolution of EU Fiscal and Economic Policy Coordination under the EU Treaties, T0 to T2

T0 (Maastricht Treaty), based on IGC 1990–1991	Treaty bases for EU economic policy coordination and surveillance: Article 103 TEC and Article 189c TEC* (i.e., the co-operation procedure); Measures and policies developed: SGP starts in 1997; Council Regulation (EC) 1466/97 addresses the issue of strengthening surveillance of budgetary positions, surveillance and coordination of economic policies. Start of national submission of the SCPs	
T1 (Amsterdam Treaty and Nice Treaty)	T1a (Amsterdam Treaty, based on IGC 1996)	T1b (Nice Treaty, based on IGC 2000)
	Treaty bases: Article 99 (ex Article 103) TEC and Article 252 (ex Article 189c) TEC (i.e., the co-operation procedure); no changes introduced by the Amsterdam Treaty to the two relevant Articles of the Maastricht Treaty; Measures and policies developed: Lisbon Strategy launched in March 2000	Treaty bases: Article 99 and Article 252 TEC; no changes brought by the Nice Treaty to the two relevant Articles of the Amsterdam Treaty and the earlier Maastricht Treaty; Measures and policies developed: (a) re-launched Lisbon Strategy in 2005; introduction of the practices of NRPs and CSRs; (b) SGP reforms in 2005, including Council Regulation (EC) No 1055/2005 amending Regulation (EC) No 1466/97
T0 to T1a/T1b: Assessment of Analytical Factors (1)–(9)		
(1) Shifts in domestic conditions	Yes	Yes
(2) Sunk costs (micro-level adaptations)	No; The SGP for budgetary surveillance and economic policy coordination was not in place when the 1996 IGC took place; the factor of micro-level adaptations was absent. Policy coordination, however, became an important factor for economic prosperity. Switching away from the idea of policy coordination encompassed costs	Yes; SGP rules and Lisbon Strategy are seen as beneficial and necessary measures to promote (micro-level) economic growth within the EU
(3) Accumulated policy constraints	No; economic policy coordination under the BEPGs and the EGs are not legally binding	No; no legally binding decisions possible on the issues concerned
(4) Heavily discounted or unintended effects	No; relevant legal bases are not changed; EU member states’ control over relevant EU policies is not reduced; EU institutions are not able to issue legally binding decisions on EU member states	No; legal bases for the issues concerned are not changed; absence of legally binding decisions remains; hence, EU member states’ control over relevant EU policies is not reduced
(5) Changed preferences of Member States	No; Amsterdam Treaty renumbers ex Articles 103 and 189c TEC; but contents are not changed	No; no changes made to the two relevant Articles taken over from the Amsterdam Treaty; co-operation procedure remains unchanged; hence power and competencies of the supranational institutions remains unchanged compared to the TEU
(6) New powers and preferences for supranational institutions	No; legal basis and co-operation procedure to adopt the measures related to economic policy coordination and fiscal surveillance is not changed compared to TEU	
(7) New decision-making rules and policy competences at the European level		
(8) Reinforcement between actors’ preferences and the rules that bind them	Yes; although there were no binding rules for the issues at stake, the SGP had established a given equilibrium between EU institutions and EU member states; launch of the Lisbon Strategy reflected preferences for enhanced co-operation between member states	Yes; although there were no binding rules for the issues at stake, a given equilibrium between EU supranational surveillance and EU member states’ ultimate decisions on their budgetary plans and economic policies was established and strengthened. The SGP reforms and the re-launched Lisbon Strategy reflected reinforced preferences of governments and EU institutions
(9) An increasing unlikeliness to change the rules codified at t endogenously	Yes; SGP was continued without changes	Yes; though the SGP was amended, the aim was to strengthen SGP rules as well as EU economic governance as a whole; basic principles entailed in the SGP were not adapted, demonstrating the ‘stickiness’ of SGP rules and a decreasing prospect to change or radically break away from the SGP provisions
T2 (Lisbon Treaty, based on IGC of 2007)	Treaty bases: Article 121 of the Treaty on the Functioning of the European Union (TFEU) *) (amending ex Article 99 TEC) and Article 294 TFEU (ex Article 251 TEC) (i.e., the co-decision procedure / ordinary legislative procedure (OLP)) Measures and policies developed: (a) Europe 2020 launched in March 2010, based on the lessons and experiences gained based on Lisbon Strategy; (b) ES implemented on 1 January 2011, codified into EU law in December 2011; (c) new coordination and monitoring elements (e.g., AMR of MIP) added into the ES procedure, particularly by the ‘six-pack’ and the ‘two-pack’ reforms of the SGP	
T1a/T1b to T2: Assessment of Analytical Factors (1)–(3)		
(1) Changes to member state preferences	Member state preferences changed from co-operation procedure to the OLP to codify relevant measures into EU law. Preference for greater role for the EP and the European Commission suggested (more democratic control and enhanced willingness to strengthen economic policy coordination and surveillance of fiscal performance on the supranational level). Presence of factor (2) at time T1b (Nice Treaty), absent at time T1a (the Amsterdam Treaty) reflects preference changes, while mechanism (4) of the posited path dependence model seems absent	
(2) Changes in member state bargaining power	EU member states essentially agree on the need to strengthen the framework of economic and fiscal governance. Agreement largely by consensus rather than reflection of given power distributions. Discussion over member state bargaining powers not deemed necessary, moreover, due to the negation of (3) and (4) at time T1b (Nice Treaty)	
(3) Power of other actors	Treaty changes made to ex Article 99 TEC; prescribed more responsibilities for the European Commission and the EP; relevant legislative procedure shifted from co-operation procedure to OLP. Thus, negation of (4) at time T1b (Nice Treaty) appeared inadequate to account for changes in the power of other actors at time T2 (Lisbon Treaty). But essence of equilibrium between the EU institutions and member states, since adoption of preventive arm of SGP in 1997, remained unchanged (due to the non-binding decisions in this domain). Redefined role of the EU institutions (3) can be explained by T1 variables of (2), (5), (8), and (9)	

^*^Before the Lisbon Treaty, the abbreviation TEC (Treaty establishing the European Community) was often used after a TEU Article of the first pillar of the EU (i.e. the Community pillar), distinguishing it from TEU articles of the second pillar (i.e., the Common Foreign and Security Policy) and the third pillar (i.e., police and judicial co-operation in criminal matters). Lisbon Treaty abolished three-pillar structure of the EU, with the TEC becoming the TFEU (while the TEU retains the same label)

Source: Adapted from Pan (2015, 2018), based on an analysis of EU Treaty provisions, assessing twelve potential indicators contained in the HI three-stage analytical model

Table 2 shows what these changes were, resulting from intergovernmental conferences and related Treaty changes. Developments from T(0) to T(1) capture changes of explanatory factors from the Maastricht Treaty to the Amsterdam and the Nice Treaty and continuing to changes between T(1) to T(2), based on the three analytical factors presented above.

As Table 2 shows, our historical-legal analysis does not provide evidence for causal effects for some of the indicators. Most notably, the respective assumptions related to factors (3), (4), (5), (6) and (7) are not discovered, partially countering assumptions integrated into the three-step analytical model specified above. This could be a result, however, of the relevance of absent variables, or alternatively, the ES case, in the framework of overall post-crisis economic governance in the EU, might constitute a special case. But nonetheless, we do not find sufficient empirical support for H2.

Given the results of our ES case study, exploring changes in legal provisions and respective criteria between rounds of ‘grand bargains’, we would like to offer thoughts on potential modifications, alterations and updates to the theoretical sequences presented above. First, it seems that unintended consequences, after the initial (intergovernmental) decisions have been taken, appear notably in the framework of policy areas and measures with legally binding powers (i.e., where national control over EU policy is constrained). Second, based on our findings as displayed in Table 2, we find that from the indicators included in the analysis, factor two (sunk costs) tended to exert decisive influence on member state preferences; by comparison, factors one (shifts in domestic conditions) and four (heavily discounted or intended effects), did not seem to matter much. Third, as our case study demonstrated, EU institutions (particularly the European Commission) tended to be assigned more functional tasks, in essentially a neo-functionalist logic.

The ES has developed into an important post-crisis EU policy coordination and surveillance mechanism. It been criticized as a main feature of ‘austerity’, but clearly introduced timelines in terms of enhanced EU economic and fiscal policy coordination and surveillance.

Arguably, the political failure to take a big constitutional step with the establishment of an actual European Constitution, aiming to further achieve an ever-closer union, might be seen as the main reason why member states were highly reluctant, after the global financial crisis of 2007–2008 and the Greek sovereign debt crisis of 2010, to initiate a revision to the Lisbon Treaty in the fiscal-economic realm. Such a revision would have aimed to tighten supranational control over member states having large macroeconomic, financial and fiscal imbalances. In addition to this, the North–South and Core-Periphery gap that had developed within the eurozone made it politically unattractive to develop a new EU Treaty which would have to pass referenda in several creditor as well as debtor countries. This reluctance restricted the available policy solutions to the crises to what was feasible within the context of the Lisbon Treaty and, hence, showed path dependency. Clearly, path dependency was driven by both political and economic factors and circumstances.

Member states had to find a compromise on a tighter EU economic governance framework, which would preserve the institutional balance, especially through secondary legislation. In contrast, European institutions favored more path-breaking reforms. For example, the European Central Bank (ECB) called for a 'quantum leap' in economic governance to strengthen the institutional foundations of the Economic and Monetary Union (EMU) (see Trichet 2010; European Central Bank 2011).

Arguably, the Lisbon Treaty opened the door for Eurozone countries to adopt measures for themselves, not applying to non-eurozone members of the EU (see TFEU, Article 136). Clearly, this accentuated the trend towards a two-speed EU, as article 136 paragraphs 1 and 2 enabled the eurozone countries to strengthen coordination and surveillance of budgetary discipline and to set out economic policy guidelines for themselves. They also used this opportunity to design a specific sanctions mechanism for repeated non-compliance with the rules of economic governance. As the Lisbon Treaty set the stage, however, this new sanctions mechanism again confirms path dependency.^2^

## Further developments

The possibility of sanctions for Euro area countries in breach of the European deficit and debt criteria was already in place with the introduction of the SGP in 1997. Yet, at the time, the sanctioning mechanism was firmly under the control of the intergovernmental institutions of the EU, as the European Commission’s recommendation of sanctions had to be approved by a qualified majority vote (QMV) in the Council of the EU before being accepted. As a result, sanctions never were imposed under the 1997 procedure even when countries flagrantly breached the imposed guidelines.

This was changed in 2011 when the Fiscal Compact and the Six Pack instituted a ‘reverse qualified majority vote’ (RQMV) procedure (e.g. Essl and Stiglbauer 2011) and created a more automatic sanctions mechanism, cementing the decisive role of the European Commission in the Excessive Deficit Procedure (EDP) and to a lesser extent, the Excessive Imbalances Procedure (EIP). The EDP has a similar institutional construction, using information provided in the ES to assess if member states breached the EDP’s indicators. The Commission notifies the Council on the breach, which then gives recommendations to the member state in question. In the case of inadequate or non-action by this state, the European Commission proposes financial sanctions up to 0.2 percent of the country’s GDP, which is automatically accepted if not for a RQMV by the Council to reject the sanctions. Taking in account such innovations and their possible implications somewhat nuances the discussion on the ES seen from an HI perspective.

To understand the wider implications of the ES, it is notably important to look at the EIP and take the innovations provided by the SGP’s corrective arm into account, namely the EDP. These instruments are inherently tied to—and rely on—the ES. The EIP is a part of the preventative arm of the Macroeconomic Imbalances Procedure (MIP), which aims to identify, prevent and address potentially harmful macroeconomic imbalances in the EU; these are monitored as part of ES, particularly through the Alert Mechanism Report (AMR). The AMR consists of a scoreboard based on a number of indicators; when it records a member state producing values above or below the ‘healthy’ thresholds, the European Commission conducts further analyses. This in turn leads to a discussion in the Council of the EU and the Eurogroup. In the case that the Commission detects alarming macroeconomic imbalances, it advises the Council to issue recommendations for corrective action and the EIP is triggered. Once the EIP has been launched, the targeted member state needs to submit a corrective plan within a specified deadline. Only if it is found to be in contravention of the EIP, however, ‘political sanctions’ may be imposed, including joint talks between the institutions and extensive surveillance or reporting procedures. When a Eurozone country is found to be in contravention of the EIP, ‘financial sanctions’ can be imposed: an annual fine of 0.1 percent of its national GDP. The imposition of sanctions is proposed by European Commission; if the Council of the EU does not reject the Commission’s proposition by QMV within ten days, the sanctions are automatically accepted.

Seikel (2016) and Dehousse (2016), for example, argued that such reforms fundamentally altered the power dynamics between the European Commission and the Council, constituting a shift towards a ‘quasi-automatic’ sanctions mechanism and changing the discussion from voting on a recommendation of the Commission to ‘overruling’ its recommendations. As such the European Commission had become the prime agenda setter and held a monopoly in terms of the right of initiative, while member states were forced to choose between the proposal of the agenda setter or to overrule it. Seikel (2016) shows, using a spatial model that rejection of the sanctions under RQMV has become much more unlikely and that as such, this innovation has clearly strengthened the European Commission’s role. Further evidence could be provided by the new position of the European Commission on economic governance in actions taken by France and by Italy. After scrutinization of the Italian budget in 2018, moreover, the Italian state was asked by the European Commission to revise its budget in line with the relevant criteria. Italy first refused to do so and thereby was under threat of the EDP being triggered. After extensive negotiations between Italy and the European Commission, the budget was revised in line with the Commission’s recommendations (Fortuna 2018). A similar discourse on the centrality of the European Commission took place in view of France’s possible breach of the debt criteria in response to measures taken to appease the ‘Yellow Jacket’ movement. The breach triggered a reaction by French Commissioner Pierre Moscovici and intense discussion in European media (Valero 2018).

Given the changed power dynamics between the European Commission and EU member states on the imposition of sanctions, which very much paralleled discussions on whether ‘austerity’ measures are useful or not in view of overall economic development, the discussion on ‘path dependency’ versus ‘critical junctures’ could be reopened. The possible fundamental shift in the power dynamics between the European Commission and EU member states could signify more of a ‘critical juncture’. However, the limited scope of the EIP and EDP’s sanction mechanism and the fact that it was already cemented in the 1997 SGP rather suggests a case of ‘path dependency’. This argument has been supported by Dehousse (2016) who refers to the fact that no quantum leaps on policy were made, but rather that the decision of RQMV was shaped in a risk adverse environment spurred by urgency and the need to show ‘credible commitments’ to the outside world. In this light, the RQMV innovation seems rather a continuation of the past than the development of a new ‘branching point’. The lack of a ‘quantum leap’ has also been emphasized by Koester et al. (2013).

More recent discussions on the 2011 reform suggest or confirm a number of different possible ‘unintended consequences’. Savage and Howarth (2018), for example, show that the ES has strengthened the European Commission’s position to an even greater extent than conceptualized during the 2011 negotiations. They state that the European Commission’s request for better data and continued accumulation of economic information might put it at an ‘unfair’ advantage in comparison to the member states in negotiations on EU economic governance. Savage and Verdun (2016) find a similar strengthening in professionalism and data access when it comes to the European Commission’s Directorates-General. This changing dynamic where the European Commission ‘holds the cards’ on EU post-crisis economic governance also shows in the European Commission’s review of the SGP, whereby it reinterpreted the legal code as established in 2015 to better suit its needs. This demonstrates that the 2011 reform further expanded the European Commission’s potential for autonomous action (European Commission 2015; Seikel 2016). Our paper does not provide a judgment on such changes, but does suggest these might indeed constitute ‘unintended consequences’ of the 2011 reform, i.e. coincidental side-effects of a path-dependent evolution. Therefore, they lend credit to a ‘path dependency’ argument. In recent literature, however, alternative interpretations regarding power relations have been offered, often referring to the power of creditor states as a central explanatory factor for (deliberate) institutional choices (e.g. Schimmelfennig 2014; Seikel 2019). Accordingly, the increase of supranational powers does not seem to be a mere by-product of other dynamics.

A last note on the ‘unintended consequences’ is that the use of RQMV could lead to similar patterns of action in other EU integration areas. According to de la Porte and Heins (2015), for example, such discussions might lead to the adoption of RQMV in more areas the EU is engaged in, and with this, constitute additional ‘unintended consequences’. Lastly, the three-step analytical model applied to the ES, based on a series of indicators, showed in our case study that several variables were unaccounted for in the end. Focusing on the 2011 reform and the wider context of the ES, the three-step analytical model might prove to be a better tool in understanding the evolution of EU economic governance, as the aforementioned issues demonstrated that there possibly were (a) multiple unintended effects (T1, indicator 4); (b) accumulated policy constraint given the debt criteria (T1, indicator 3) and new decision-making rules (RQMV) and policy competences at the European level (T1, indicator 7). While our hypotheses were not fully confirmed, evidence for these sub-aspects was found.

The ES is only one case that can be explored in detail based on HI assumptions and similarly, it is not the only theoretical framework applicable to explore this case. Of course, the ES is only one element in the overall EU economic governance regime applicable and with this, provides a partial analysis of EU post-crisis surveillance and coordination procedures.

Finally, another new element of euro area multilateral surveillance is that the country-specific recommendations under the European Semester now also include a recommendation addressed to the euro area as a whole, to further improve economic policy coordination. A more path-changing feature, however, was the addition of a new paragraph 3 to Article 136 of the Lisbon Treaty – which did not need to get approval in domestic referenda – allowing the eurozone countries to establish the European Stability Mechanism (ESM) as an institutional innovation. But as the ESM is based on an intergovernmental treaty (outside the EU framework), it arguably keeps the national-national power relations intact.

## Conclusions

Our case study focused on the ES has assessed, based on the HI theoretical framework and using a range of indicators, whether the ES constituted a ‘critical juncture’ in the sense of a radical departure from an earlier path of European economic and fiscal governance taken, or whether it was largely a result of a process of ‘path dependence’. We formulated two hypotheses and carried out a historical-institutional analysis based on the legal provisions contained in Treaty reforms, ranging from the Maastricht Treaty via the Nice and Amsterdam Treaties to the Lisbon Treaty. In the scheme of the three-step analytical model applied, T(0) reflected the situation encompassed in the TEU (Maastricht Treaty), T(1) the Nice and Amsterdam Treaty provisions and T(2) the respective contents of the Lisbon Treaty. Exploring the respective Treaty articles and their respective formulations, we created a table demonstrating adaptations over time. Based on the results of our analysis, using specified indicators, we concluded that the ES was a result of path dependence or ‘incremental change’. The distribution of power in either a member state to member state perspective (horizontal power distribution) or a comparison between the power allocation between EU member states and the supranational level (vertical power distribution) was not seen as having been strongly affected, providing further evidence for the lack of a critical or path-changing juncture.

While supranational institutions in the EU (including the ECB) called for a ‘quantum leap’ in terms of new patterns of EU fiscal governance, the 2011 SGP and 2012 Fiscal Compact only represented small steps ahead on an already existing path (e.g., Koester et al. 2013; Dehousse 2016). Supranational preferences for path-changing reforms in response to the crises (e.g., Van Rompuy 2012) and how EU institutions could sketch the road towards a 'genuine EMU' which would change the national-supranational balance of power relations for the eurozone, were not answered by the political process of negotiations among member states. Euro area countries only accepted a subset of these proposals outside the realm of economic governance (particularly the idea to establish a Banking Union, which transferred national supervisory responsibility for systemic banks to the ECB).

Nonetheless, arguably, later steps and development do contain aspects such as a stronger agenda-setting power by the European Commission, which might be seen as a ‘critical juncture’ and may have reflected ‘unintended consequences’ of earlier decisions taken. The 2011 reforms to the economic and fiscal policy regime of the EU (also see European Central Bank 2011) clearly built on the earlier SGP provisions (including its ‘preventive arm’), but constituted a continuation of related fiscal and budgetary measures. The results of the ES process are more binding, however, when the EDP or the EIP of the corrective arm of the SGP are activated and with this, the competences of the European Commission increased.

As our case study demonstrated, however, the establishment of the ES does not entirely fit a HI three-step (T0-T1-T2) approach. This can be due to many decisions within the ES regime not being legally binding. Moreover, our case study revealed that out of 12 potential factors (indicators) suggested, micro-level adaptations or ‘sunk costs’ (indicator 2), as well as switching costs from the rules codified at time t (T0), turned out to have been decisive as regards member state preference formation and thus for the adoption of respective fiscal and economic coordination and surveillance mechanisms. The ‘sunk costs’ (and switching costs) found also confirm an indispensable role of collective policy coordination and surveillance at the EU level. Overall, we do not observe major ‘crisis-driven integration’ in this case.

HI was found to have been particularly useful to guide the analysis based on a range of relevant indicators. As a bridge between claims made between intergovernmentalist and neo-functionalist ‘camps’, it provides a useful angle to explore in more detailed ways processes and developments of EU post-crisis economic governance.

## Footnotes

1 We wish to thank two anonymous reviewers for most helpful comments and feedback provided on an earlier version of our paper. 

2 We thank one of our reviewers for highlighting these important aspects in convincing ways.
